# Editorial: AI innovations in neuroimaging: transforming brain analysis

**DOI:** 10.3389/fmed.2025.1755373

**Published:** 2026-01-13

**Authors:** Shyam Bihari Goyal, Deepti Deshwal, Pardeep Sangwan

**Affiliations:** 1 School of Computer Science and Engineering, Chitkara University, Rajpura, India; 2 Department of Electronics and Communication Engineering (ECE), Maharaja Surajmal Institute of Technology, New Delhi, India

**Keywords:** brain-inspired computing, EEG signal classification, fMRI pattern analysis, neuroscience, recurrent neural networks (memory + sequences)

Over the last decade, artificial intelligence (AI) has transformed nearly every branch of medical imaging, but its impact on neuroimaging has been particularly revolutionary (1–5). From automated segmentation of magnetic resonance imaging (MRI) data to deep learning–assisted disease prediction, AI techniques—especially machine learning (ML), deep learning (DL), and emerging quantum computing paradigms are reshaping how clinicians interpret the human brain. These computational advances are accelerating the diagnosis of neurological disorders, optimizing patient management, and opening new frontiers in personalized medicine (6–10).

The Research Topic “*AI Innovations in Neuroimaging: Transforming Brain Analysis*” brings together a diverse collection of studies that harness advanced algorithms and hybrid models to address key clinical challenges in brain analysis, ranging from tumor classification and stroke detection to autism spectrum disorder (ASD) assessment and schizophrenia identification. Each contribution underscores how AI, when aligned with clinical neuroimaging, can enable faster, non-invasive, and highly interpretable diagnostics.

This Research Topic presents 11 articles that collectively highlight the breadth of AI-driven neuroimaging research. The contributions span a wide range of applications from brain tumor detection and stroke prediction to epilepsy monitoring and autism diagnosis demonstrating how interdisciplinary advances are transforming precision medicine and neuroscience.

Among the notable contributions, Priyadharshini et al. introduce *QBrainNet*, a hybrid quantum-classical neural network that leverages quantum superposition and entanglement to improve stroke prediction accuracy to 96%, outperforming traditional CNN-based approaches. By combining quantum feature extraction with variational quantum circuits, this model demonstrates the transformative role of quantum-assisted intelligence in medical imaging. In another important development, Cüce et al. propose a hybrid deep learning radiomics framework that analyzes cerebrospinal fluid (CSF) signals in central nervous system infections (CNSIs). Their approach accurately identifies infection-related CSF alterations on MRI scans, offering a promising non-invasive alternative to lumbar puncture, traditionally the gold standard in CNS infection diagnosis.

Broadening the perspective beyond imaging, Farhah et al. present a Double Deep Q-Network (DDQN) model to identify ASD traits from social media text, demonstrating how digital footprint analysis can complement neuroimaging by capturing behavioral and emotional cues indicative of neurodevelopmental disorders. Similarly, Yuan et al. apply a robust multi-task feature selection strategy with counterfactual explanations to identify schizophrenia-related functional brain networks from resting-state fMRI data, enhancing both classification accuracy and clinical interpretability. These studies illustrate how AI-driven behavioral and cognitive analysis extends neuroimaging beyond the scanner to the digital and functional realms of brain health.

Advancing the field of brain tumor detection, Han et al. modify the YOLOv11 architecture by integrating novel attention mechanisms and a hybrid loss function (HKCIoU), achieving improved accuracy and reduced computational cost—an essential step toward real-time tumor detection in clinical environments. Naeem et al. complement this effort with a lightweight CNN tailored for small MRI datasets, achieving 99% accuracy and proving that data-efficient deep learning can yield high reliability even with limited samples. Alsubai et al. further expand diagnostic scope by combining transfer learning and explainable AI (XAI) for multi-disease MRI classification, accurately identifying both brain tumors and Alzheimer's disease across datasets. The integration of SHapley Additive exPlanations (SHAP) ensures transparency, allowing clinicians to visualize model reasoning. Meanwhile, Chen et al. introduce a Mixed Local and Global (MLG) model that fuses CNN and Transformer architectures through a gated attention mechanism. By integrating fine-grained and contextual features, their model achieves near-perfect accuracies (99.02% and 97.24%) and sets a new benchmark for hybrid architectures in neuroimaging.

Moving from structural MRI to electrophysiological data, Al-Adhaileh et al. employ EEG-based ML and DL frameworks for epileptic seizure detection, achieving an exceptional 99.9% accuracy using Random Forests. This demonstrates the capability of non-invasive EEG-based AI systems for reliable real-time seizure monitoring. Complementarily, Yuan et al. enhance feature interpretability in schizophrenia detection by applying counterfactual modeling to identify functional connectivity abnormalities, providing a neurobiological rationale behind model predictions.

In the domain of multimodal neuroimaging, Chandrasekaran et al. propose a powerful ensemble model combining VGG19 and Bidirectional LSTM with LightGBM for MRI-based brain simulations, achieving 97% accuracy and an AUC of 0.997. This hybrid design demonstrates how spatial and temporal feature fusion can improve diagnostic performance while supporting sustainable healthcare AI, a crucial step toward scalable clinical deployment. Collectively, these contributions highlight the evolution of AI in neuroimaging from task-specific models toward integrated, interpretable, and efficient systems capable of supporting real-world clinical decision-making. Ciftci et al. present a dual-model AI framework that synergistically combines clinical analytics and neuroimaging to improve Alzheimer's disease diagnosis. An Artificial Neural Network (ANN) trained on demographic and behavioral data from 1,200 patients provides risk prediction with 87.08% accuracy, while a Convolutional Neural Network (CNN) analyzes 4,876 MRI scans to stage disease progression with 97% accuracy using explainable Grad-CAM visualizations. By integrating structured clinical features with imaging-based assessment, the hybrid system enhances both diagnostic precision and clinical interpretability, aligning with the growing trend toward multimodal, scalable, and AI-assisted neuroimaging solutions for neurodegenerative disorders.

## Emerging themes across the Research Topic

Across the 11 studies in this Research Topic, several unifying themes emerge. First, hybrid intelligence, the integration of quantum computing, CNNs, Transformers, and ensemble learning, is redefining neuroimaging accuracy and adaptability. Second, explainability has become a cornerstone of modern neuro-AI research. Through SHAP, counterfactual reasoning, and attention visualization, the models presented here strive not only for accuracy but also for interpretability, fostering clinical trust in AI-driven diagnostics. Third, the move toward data-efficient models such as lightweight CNNs and transfer learning underscores a shift toward accessibility, enabling AI adoption even in data-constrained healthcare systems.

Additionally, multimodal integration combining MRI, fMRI, EEG, and behavioral data reflects a growing recognition that brain disorders are inherently multifactorial and cannot be captured through a single data source. These multimodal approaches bridge the gap between structure and function, allowing for more holistic assessments of neurological conditions. Finally, the emphasis on sustainability and scalability ensures that emerging AI technologies can transition from research prototypes to clinical practice, empowering healthcare systems globally.
